# The neurotrophin receptor p75NTR mediates early anti-inflammatory effects of estrogen in the forebrain of young adult rats

**DOI:** 10.1186/1471-2202-6-58

**Published:** 2005-09-12

**Authors:** Vanessa L Nordell, Danielle K Lewis, Shameena Bake, Farida Sohrabji

**Affiliations:** 1 Department of Human Anatomy and Medical Neurobiology, Texas A&M University System Health Science Center College of Medicine, College Station, TX, 77843 USA

## Abstract

**Background:**

Estrogen suppresses microglial activation and extravasation of circulating monocytes in young animals, supporting an anti-inflammatory role for this hormone. However, the mechanisms underlying estrogen's anti-inflammatory effects, especially *in vivo*, are not well understood. The present study tests the hypothesis that anti-inflammatory effects of estrogen are mediated by the pan-neurotrophin receptor p75NTR. Previously, we reported that estrogen attenuated local increases of interleukin(IL)-1β in the NMDA-lesioned olfactory bulb, while further increasing NGF expression.

**Results:**

The present studies show that this lesion enhances expression of the neurotrophin receptor p75NTR at the lesion site, and p75NTR expression is further enhanced by estrogen treatment to lesioned animals. Specifically, estrogen stimulates p75NTR expression in cells of microvessels adjacent to the lesion site. To determine the role of this receptor in mediating estrogen's anti-inflammatory effects, a p75NTR neutralizing antibody was administered at the same time the lesion was created (by stereotaxic injections of NMDA) and specific markers of the inflammatory cascade were measured. Olfactory bulb injections of NMDA+vehicle (preimmune serum) increased IL-1β and activated the signaling molecule c-jun terminal kinase (JNK)-2 at 6 h. At 24 h, the lesion significantly increased matrix metalloproteinase (MMP)-9 and prostaglandin (PG)E_2_, a COX-2 mediated metabolite of arachadonic acid. All of these markers were significantly attenuated by estrogen in a time-dependent manner. However, estrogen's effects on all these markers were abolished in animals that received anti-p75NTR.

**Conclusion:**

These data support the hypothesis that estrogen's anti-inflammatory effects may be, in part, mediated by this neurotrophin receptor. In view of the novel estrogen-dependent expression of p75NTR in cells associated with microvessels, these data also suggest that the blood brain barrier is a critical locus of estrogen's neuro-immune effects.

## Background

Estrogen replacement in adult female rats is neuroprotective for a variety of experimentally induced neural injury models, such as ischemia [1-3], toxins [4-6], and forebrain transections [7]. Estrogen is an important modulator of the neural inflammatory response, principally via its actions on microglia, the brain-resident immune cell. In primary cultures of microglia or in glial cell lines, estrogen pretreatment attenuates lipopolysaccharide (LPS)-induced superoxide release, phagocytic activity [8] and inducible nitric oxide synthase (iNOS) [8-11]. *In vivo*, estrogen treatment prevents the activation of microglia and recruitment of monocytes following injections of LPS [6], and attenuates expression of the inflammatory cytokine interleukin (IL)-1β following excitotoxic lesions [5].

The mechanisms by which estrogen exerts an anti-inflammatory effect, especially *in vivo*, remain poorly understood. In this report, we test the hypothesis that estrogen's anti-inflammatory effects are mediated via the neurotrophin family of growth factors and receptors. Members of this family include nerve growth factor (NGF), brain-derived neurotrophic factor (BDNF) and neurotrophins (NT)-3 and 4/5. Although first identified by their potent effects on neuronal survival and differentiation, NGF and BDNF are widely distributed and have a broad range of effects. BDNF, for example, is widely synthesized in the vascular system [12-15] and NGF is synthesized in immune cells (for review see [16]). Local NGF expression increases dramatically following a variety of injurious stimuli [17-19] and may prevent toxic effects of other inflammatory cytokines [20].

P75NTR, the pan neurotrophin receptor which binds NGF and all the known neurotrophins, also increases following neuronal injury [21,22]. This increase may be associated with glia [23] and endothelial cells [24]. Several studies have shown that NGF, when bound to p75NTR, initiates apoptosis in several cell types (reviewed in [25]) and recent evidence suggests that pro-NGF may preferentially bind p75NTR and initiate apoptosis [26,27]. P75NTR-NGF interactions have also been implicated in suppressing the immune response by inhibiting the induction of major histocompatibility complex (MHC) class II proteins [28] and by suppressing the transmigration of circulating immune cells to the brain [24,29].

Although classified as a neurotrophin receptor, p75NTR may also be considered a member of the tumor necrosis factor receptor (TNFR) family, due to the presence of specific domains called TRAF-interacting motifs or TIMs [30]. TRAF's are TNFR-associated factors, and recruitment of these factors by TIM-containing members of the TNFR family results in the activation of multiple signaling pathways such as NFkB, JNK, ERK and PI-3K, which lead to immune and inflammatory responses as well as cell survival and differentiation (for review see [31]). P75NTR has been shown to bind recruitment molecules such as the interleukin-receptor associated kinase (IRAK) [32], which are typically recruited by IL ligand-receptor complexes to activate NFkB and to initiate a pro-inflammatory cascade. Although the downstream consequences of p75NTR-IRAK interactions are not well known, one possibility is that p75NTR competes with IL-1β for this molecule, and may thus attenuate the inflammatory cascade initiated by the interleukins.

The present study was designed to determine the mechanism of estrogen's anti-inflammatory effect in lesioned forebrain of young adult females. Specifically, we examined whether estrogen interacted with the pan-neurotrophin receptor p75NTR to suppress the inflammatory cascade. Following brain injury, there is a rapid synthesis and release of inflammatory cytokines such as IL-1β. These cytokines activate signaling intermediaries such as NFkB and JNK to initiate the transcription of downstream inflammatory genes. We tested the effects of bulb lesions on key portions of this cascade (IL-1β expression, JNK activation, PGE_2 _and MMP-9 expression) in animals that were given either estrogen or control pellets. The role of p75NTR was evaluated by using a neutralizing antibody to this receptor, injected into the lesion site. In the present study, we used NMDA-induced olfactory bulb lesions, which we have previously shown results in an inflammatory response as indicated by microglial activation and increases in IL-1β [5]. Here we report that olfactory bulb lesions to ovariectomized females increase p75NTR expression, and receptor expression is further enhanced in females that received estrogen treatment. Specifically, estrogen treatment stimulates p75NTR expression in cells associated with microvessels. Antibody experiments reveal that while estrogen treatment suppresses the inflammatory cascade resulting from bulb lesions, blocking the p75NTR receptor completely abolishes estrogen's effects on the early and late markers of the inflammatory cascade, but has no effect on these markers in controls (non-estrogen treated animals). These data support the hypothesis that estrogen's anti-inflammatory effects are mediated through p75NTR. In view of our recent studies that estrogen reduces extravasation of dye from circulation to brain tissue [33] and the current evidence that estrogen stimulates p75NTR expression in the wall of microvessels, we propose that early in the course of brain injury, estrogen pretreatment suppresses the inflammatory cascade by affecting the blood brain barrier.

## Results

### Confirmation of estrogen treatment

Plasma estradiol levels were measured from trunk blood collected at termination (approximately three weeks after pellet implantation). Ovariectomized animals replaced with an estrogen-containing pellet (E2) had average plasma estradiol levels of 51.66 ± 4.19 pg/ml, typical of levels seen at proestrus in this animal. The average weight gain as a result of estrogen treatment was -6.24 ± 2.1 g. In contrast, ovariectomized, control pellet-replaced animals (OVX) had low estradiol levels (9.1 ± 0.49 pg/ml) with a corresponding weight gain of 57.5 ± 2.76 g.

### Confirmation of anti-p75NTR antibody treatment

In the present studies, the effectiveness of anti-p75NTR antibodies was tested *in vivo *by measuring caspase-3 activity. Caspase-3, an effector caspase that is proximal to the apoptotic cell death cascade [34], was used as a marker since p75NTR has been implicated in apoptotic cell death. Caspase-3 activity was significantly reduced (by 40%, p < 0.05) in animals that received NMDA+anti-p75NTR antibody (0.73 ± 0.06 pmol AMC liberated/min at 37°C/mg protein) as compared to animals that received NMDA+preimmune serum (1.23 ± 0.04 pmol AMC liberated/min at 37°C/mg protein).

Specificity of the actions of the p75NTR antibody was not tested directly, by the use of non-specific antibody or antibody to an unrelated protein. However, as described below, the actions of the antibody were seen only in estrogen-treated animals and not in control-treated animals. Furthermore, the actions of the antibody reversed estrogen's actions on inflammatory markers (see Figures 3, 5, 6 and 7), but did not reverse estrogen's effects on the neurotrophin NGF (see Figure 8).

### P75NTR regulation following olfactory bulb lesions

**Figure 1:**
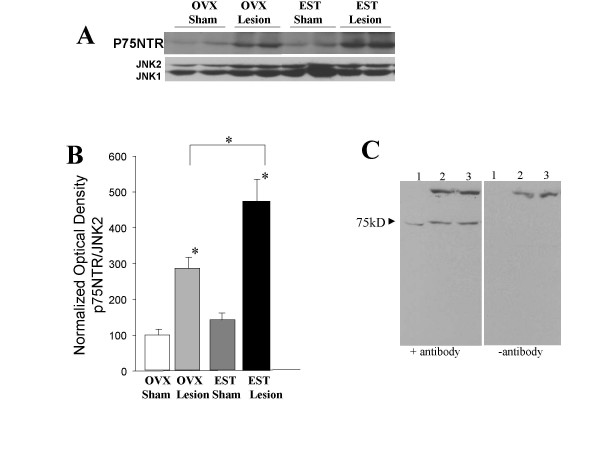
P75NTR regulation following olfactory bulb lesions: P75NTR expression was determined by Western blot assays (A) and receptor expression was quantified and normalized to a loading control (JNK) in the histogram (B). P75NTR was increased in lesioned animals, and estrogen treatment for 3 weeks prior to the lesion further enhanced the expression of this receptor. Two representative examples are shown of each treatment condition in the Western blots. Bars represent mean ± SEM, n = 6 per group. (C) Specificity of p75NTR antibody: The antibody used in this Western assay recognizes a size appropriate band at 75 kD, which is not seen when the membrane is probed in the absence of the primary antibody. Lane 1: PC12 cell lysate, Lanes 2,3: olfactory bulb lysate. Key: OVX Sham: Ovariectomized female replaced with a control pellet, with sham injection, OVX lesion: ovariectomized female replaced with a control pellet, with NMDA injection, Est Sham: ovariectomized female replaced with an estrogen pellet, with sham injection, Est Lesion: ovariectomized female replaced with an estrogen pellet, with NMDA injection. *: p < 0.05.

P75NTR expression in olfactory bulb lysates was determined by Western blot assay (Figure 1A), and the quantitated signal, normalized to JNK, is shown in a histogram in Figure 1B. P75NTR expression increased 3-4 fold in lesioned animals as compared to sham-injected animals (F_1,20_: 12.2; p < 0.05). Estrogen treatment further enhanced the level of lesion-induced p75NTR (F_1,20_: 4.99, p < 0.05). As reported before, this is the group where expression of the inflammatory cytokine IL-1β is attenuated by estrogen [5]. The p75NTR antibody used in Western blot assays recognized a size-appropriate band in olfactory bulb tissue and PC12 cells, a prototypic p75NTR positive cell line.

### P75NTR immunohistochemistry

**Figure 2:**
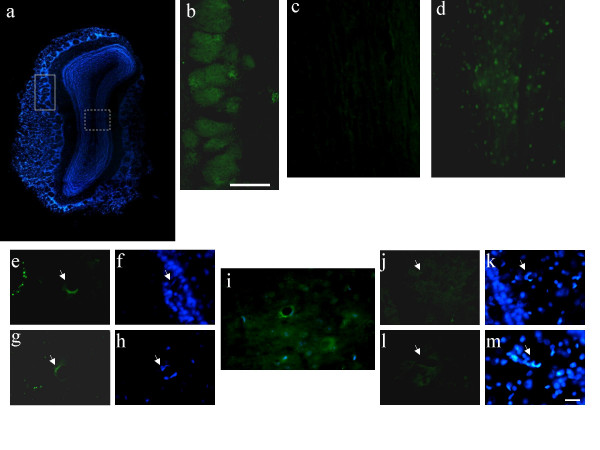
P75NTR expression in the lesioned olfactory bulb: Olfactory bulb sections from sham and NMDA lesioned animals that were either estrogen or control-pellet replaced were probed for p75NTR expression and counter-stained with a nuclear dye (DAPI). A low magnification photomontage of olfactory bulb illuminated for DAPI is shown in (a). P75NTR is normally expressed in the glomerular layer of the olfactory bulb (b), photographed from the region indicated by the solid line box in (a). P75NTR is poorly expressed in other regions of the olfactory bulb, an example of which is shown in (c), taken from the region indicated by the wire frame in (a). However, in a comparable region of the NMDA-lesioned animals, diffuse p75NTR staining is seen in cells and fibers at the injury site (d). In estrogen-treated, lesioned animals p75NTR immunoreactivity is seen in cells associated with the walls of microvessels (e; g), identified by their curved nuclei (f; h) surrounding a lumen. Arrows indicate the same cells photgraphed under fluorescein (p75NTR; e,g) or UV illumination (DAPI nuclear dye; f,h). A double-labeled cell photographed under both fluoresein and UV is shown in (i). P75NTR labeling was not seen in microvessel walls in lesioned animals that received a control pellet (j) or in sections from estrogen-replaced lesioned animals that were incubated without a primary antibody (immunohistochemical control, l). Corresponding nuclear dye images are in k and m. Magnification bar: b-d = 160 μm; e-m = 25 μm.

P75NTR expression in the olfactory bulb of sham and lesioned animals was determined by immunofluorescence (Figure 2), using the same antibody that was used for Western blot analysis. A few sections were also probed with the neutralizing p75NTR antibody. Figure 2a shows the cellular architecture of the olfactory bulb in a section stained with a nuclear dye (DAPI). With either p75NTR antibody, prominent staining was noted in the fibers of the glomerular layer of lesion and sham-injected animals (Figure 2b; taken from region indicated by solid-line box in 2a), similar to that reported by others [35]. While the central portion of the bulb was poorly immunoreactive for p75NTR in the sham-lesion animals (Figure 2c), NMDA injections resulted in bright staining in fibers and cells surrounding the lesion site (Figure 2d). Although the type of cell and fibers is not known currently, the pattern of staining did not appear to be any different in the estrogen-replaced and estrogen-deprived animals. This diffuse staining pattern likely accounts for the bulk of p75 expression observed following lesions in the Western blot analysis. The only location where p75NTR expression differed in estrogen- and control-treated lesioned animals was associated with blood vessels. P75NTR-positive staining was visible in cells lining microvessels near the lesion site in estrogen-treated animals (Figure 2e and 2g). Typically, these vessels contained one or two curved nuclei, characteristic of endothelial cells (visualized by DAPI, Figure 2f and 2h), indicating that these vessels were either capillaries or postcapillary venules. A high magnification image from a section of an estrogen-treated animal photographed under both UV and fluorescein illumination, shown in Figure 2i, depicts p75NTR (in green) localized to a cell lining the vessel wall, identified by its curved nucleus (in blue). While microvessel staining was easily seen in the estrogen-treated animals, there was virtually no p75NTR staining seen in microvessels in the control pellet replaced animals (Figure 2j; nuclear stain in 2k) and in control sections from lesioned, estrogen-replaced animals where the primary antibody was not applied (Figure 2l; nuclear stain in 2m). Photomicrographs (e) through (m) were obtained from the region indicated by the hatched box shown in 2a.

### Markers of inflammation

To test if p75NTR mediates the anti-inflammatory effects of estrogen, control and estrogen-pellet replaced animals were injected with NMDA+anti-p75NTR, and compared to sham lesioned controls and controls that received NMDA+preimmune serum. Animals were either terminated 6 or 24 h after the lesion, and olfactory bulb protein lysates were used to measure several proteins associated with the inflammatory cascade.

### IL-1β expression

**Figure 3:**
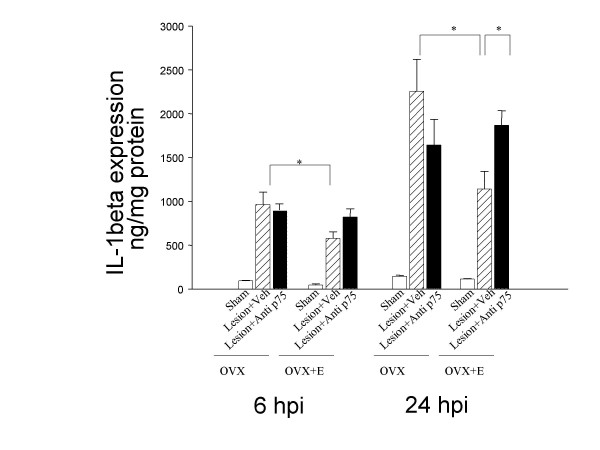
IL-1β regulation by lesion and estrogen treatment: Olfactory bulb lesions significantly increased local IL-1β expression at 6 and 24 hours post injury (6 hpi, 24 hpi). Estrogen treatment (OVX+E) attenuated IL-lβ at 6 hpi, and this effect was more pronounced at 24 hpi. At 24 hpi, anti-p75NTR treatment abolished estrogen's effects on IL-1β. Anti-p75NTR had no effect on IL-1β expression in control pellet-replaced (OVX) animals. Bars represent means ± SEM, n = 6 per group. Sham: Sham lesion, Lesion+Vehicle: NMDA injection with pre immune serum, Lesion+Anti p75: NMDA injections with anti p75NTR antibodies. *:p < 0.05.

Local levels of IL-1β, measured by ELISA assay, increased dramatically at 6 h (F_2,30_: 51.61, p < 0.05) and 24 h (F_2,30_: 39.31, p < 0.05) after NMDA injections (Figure 3). At 6 h after lesion, estrogen treated groups had slightly lower levels of IL-1β as compared to placebo groups (F_1,30_: 5.72, p < 0.05). At 24 hours after lesion, estrogen replacement (OVX+E) clearly blunted the injury-related increase in IL-1β (F_2,30_: 4.99, p < 0.05). However, neutralizing p75NTR antibodies completely abolished estrogen's effects, causing IL-1β levels to be no different from those seen in the control-pellet (OVX) replaced NMDA-lesioned group. Note that anti-p75NTR treatment had no effect on the control-replaced group.

### Regulation of IL-1β mRNA and ICE activity

**Figure 4:**
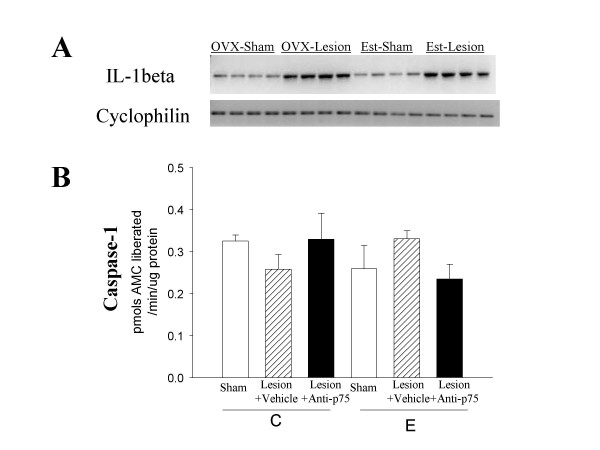
Regulation of IL-1β mRNA and the interleukin-converting enzyme: (A) mRNA from lesioned or sham injected animals, with estrogen or control pellets was reverse transcribed and amplified using primers specific for IL-1β and cyclophilin. PCR product was fractioned on an agarose gel and photographed under UV illumination. An image of these gels is shown here, with 4 representative animals from each group. At 24 h post injury, NMDA lesions increased IL-1β mRNA, however, estrogen-treated (OVX+E) animals were no different from the control-pellet (OVX) treated animals. (B) Since the mature form of IL-1β is obtained through cleavage of IL-lβ precursor protein by caspase-1, activity for this enzyme was also assayed. Neither NMDA lesions nor estrogen treatment caused any significant changes in caspase-1 activity at 24 h post injury. Histogram bars represent means ± SEM for the entire group (n = 6). *:p < 0.05.

To determine the locus of hormone action on this cytokine, IL-1β mRNA levels and the activity of the interleukin-converting enzyme (ICE or Caspase-1) was measured. As shown in Figure 4A, RT-PCR analysis indicated that the lesion substantially increased IL-1β mRNA (F_1,12_: 146.85; p < 0.05) at 24 h, however, estrogen pretreatment did not affect mRNA expression (F_1,12_: 0.01, p > 0.05). Furthermore, activity levels of caspase-1, which cleaves the pro-peptide to mature IL-1β, were not regulated by lesion or estrogen treatment (F_5,30_: 0.99; p > 0.05) at 24 h, (Figure 4B), suggesting that this pathway is not initially responsible for the increase in activated IL-1β.

### pJNK expression

**Figure 5:**
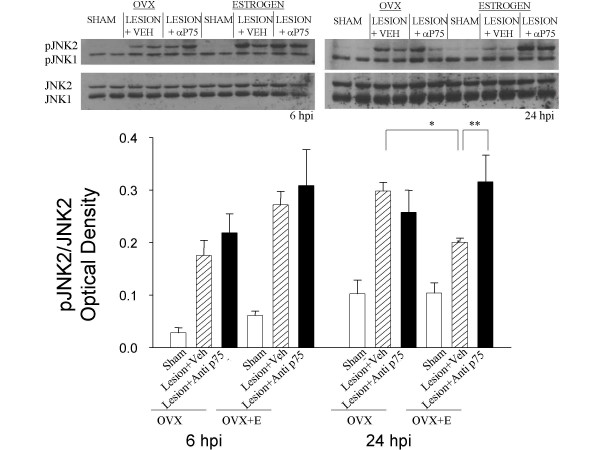
JNK activity following olfactory bulb lesions: JNK activation was measured by Western blot assay using phospho-specific (p) antibodies, and pJNK2 expression was normalized to total JNK2 protein. JNK1 was constitutively active in all cases. Virtually no pJNK2 was seen in the sham-injected controls in either control or estrogen-replaced animals. Bulb lesions significantly increased pJNK2 at 6 h after the lesion. At 24 h, pJNK2 levels were still elevated in the control-replaced (OVX) lesioned animals but significantly attenuated in the estrogen-treated (OVX+E) lesioned group. Anti-p75NTR reversed the effects of estrogen on pJNK2 activation at 24 h, although it had no effect on the control-pellet group. Two representative examples are shown from each treatment condition in the Western blot images. Histogram bars represent means+SEM for the entire group (n = 6). Sham: Sham lesion, Lesion+Vehicle: NMDA injection with pre immune serum, Lesion+Anti p75: NMDA injections with anti-p75NTR antibodies. *:p < 0.05.

C-jun kinase activation was determined using antibodies specific for the phosphorylated form of the protein (pJNK) and normalized to total JNK protein (Figure 5). Histogram shows the ratio of pJNK2/JNK at 6 and 24 hours post injury (hpi). JNK is one of the signaling molecules activated by IL-1β when the latter is bound to its receptor. As shown in Figure 5, pJNK2, but not pJNK1, was increased by lesion at 6 h (F_2,30_: 19.22, p < 0.05) and remained activated at 24 h (F_2,30_: 19.9, p < 0.05). pJNK1, on the other hand, was constitutively active, which is typical for this isoform. JNK2 has a higher affinity for c-jun [36], and activated JNK2 translocates to the nucleus, while pJNK1 generally remains in the cytoplasmic compartment [37]. Post hoc analysis indicated that estrogen treatment (OVX+E) attenuated pJNK2 activation in lesioned animals at 24 h as compared to control replaced animals. However, concurrent injection of NMDA+antip75NTR abolished estrogen's effects on pJNK2 at 24 h, such that the relative activation of this molecule was no different from that of control-treated (OVX) animals.

### Prostaglandin E_2 _(PGE_2_) levels

**Figure 6:**
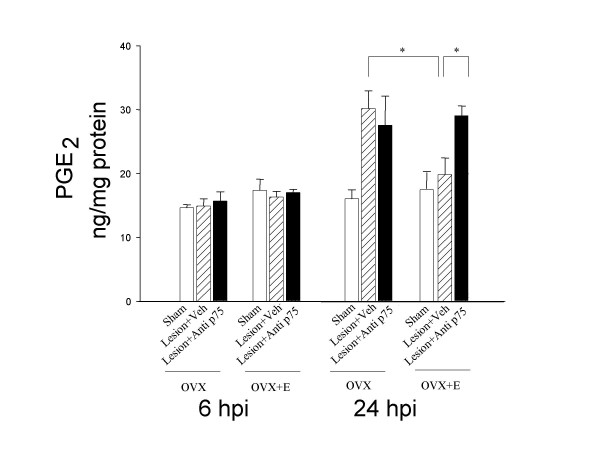
PGE_2 _levels in the lesioned olfactory bulb: PGE_2 _expression was not altered by lesion or hormone treatment at 6 h post injury (6 hpi). However, at 24 hpi, PGE_2 _expression increased 2-fold in the control-pellet (OVX) replaced lesioned groups, while this increase was not seen in the estrogen-treated (OVX+E) lesioned animals. However, anti-p75NTR treatment completely abolished estrogen's suppressive effects, making this group indistinguishable from the control pellet replaced, lesioned animals. Bars represent means ± SEM, n = 6 per group. Sham: Sham lesion, Lesion+Vehicle: NMDA injection with pre immune serum, Lesion+Anti p75: NMDA injections with anti-p75NTR antibodies. *:p < 0.05.

At 6 h after the lesion, PGE_2 _levels in the olfactory bulb were no different from sham controls (F_2,30_: 0.21, p > 0.05; Figure 6), indicating that this marker occurs later in the inflammatory cascade. At 24 hours post injury, there was a 2-fold increase in PGE_2 _levels in lesioned animals that were deprived of estrogen, while estrogen treatment completely attenuated this increase in PGE_2_. Anti-p75NTR antibodies completely eliminated estrogen's effects, causing PGE_2 _levels to rise similar to those seen in the control-replaced (OVX) animals. Note that as with IL-1β, anti-p75NTR treatment had no effect on PGE_2 _levels in the control-replaced group.

### MMP-9 activity

**Figure 7:**
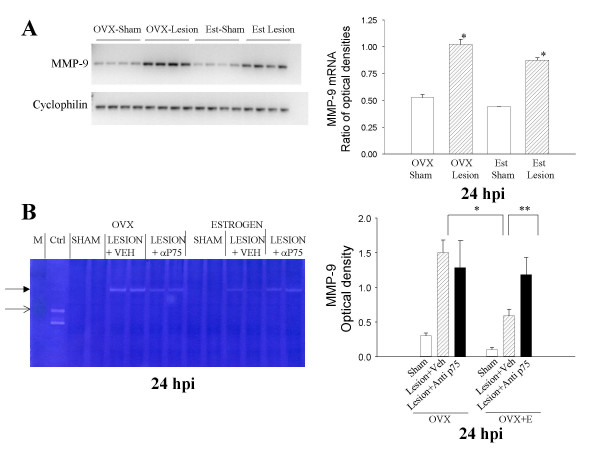
MMP-9 regulation: (A) MMP-9 mRNA was assayed by RT-PCR, and normalized to cyclophilin RNA. MMP-9 mRNA was significantly increased by lesion, and estrogen teatment resulted in a small but signficant decrease in MMP-9 mRNA expression. (B) MMP-9 activity was measured by gelatin zymography at 6 and 24 h post injury, and representative examples from each group are shown here. The location of the lytic area corresponded to the expected size of MMP-9 (96 kD), indicated by the filled-arrow head, as well as that of a positive control (conditoned media from endothelial cells). Line-arrow head indicates pre-stained protein size marker (86 kD). Virtually no MMP-9 activity was apparent in the sham-injected animals. At 24 h after lesion, olfactory bulb lesions increased MMP-9 activity, although this was attenuated in the estrogen-treated (OVX+E), lesioned animals. Estrogen-treated lesioned animals that received anti-p75NTR, however, had MMP-9 activity that was similar to that of the control-pellet (OVX) replaced lesioned animals. Histogram represents means+SEM of the measured lytic area for MMP-9 at the 24 h time point, n = 6 per group. Sham: Sham lesion, Lesion+Vehicle: NMDA injection with pre immune serum, Lesion+Anti p75: NMDA injections with anti p75NTR antibodies. *:p < 0.05.

The 96 kD metalloproteinase MMP-9 is secreted by several cell types and is activated by cytokines via the JNK/NFkB pathways. To test whether the lesion influenced expression of MMP-9, both mRNA expression of this gene and functional activation of its protein were analyzed by RT-PCR (Figure 7A) and gelatin zymography (Figure 7B), respectively. While MMP-9 mRNA expression was increased in olfactory bulb lysates 24 h after the lesion (F_1,12_: 200.32; p < 0.05; Figure 7A), estrogen treatment resulted in a small but signifcant reduction of MMP-9 mRNA (F_1,12_: 12.4; p < 0.05, main effect of hormone). Lesion-induced increases in MMP-9 mRNA were paralleled by increased MMP-9 activity at 24, but not 6 hours post injury (data not shown), as assayed by gelatin zymography. At 24 hpi, there was a significant increase in MMP-9 gelatinolytic activity as a result of the lesion (F_2,30_: 10.12, p < 0.05), and estrogen treatment attenuated MMP-9 activity in lesioned animals (F_1,30_: 21.15, p < 0.05). As with other markers of inflammation measured here, post-hoc analysis indicated that estrogen's effect on MMP-9 activity was reversed in estrogen-treated animals that received anti-p75NTR treatment (F_2,30_: 3.84, p < 0.05; Figure 7B).

### NGF expression

**Figure 8:**
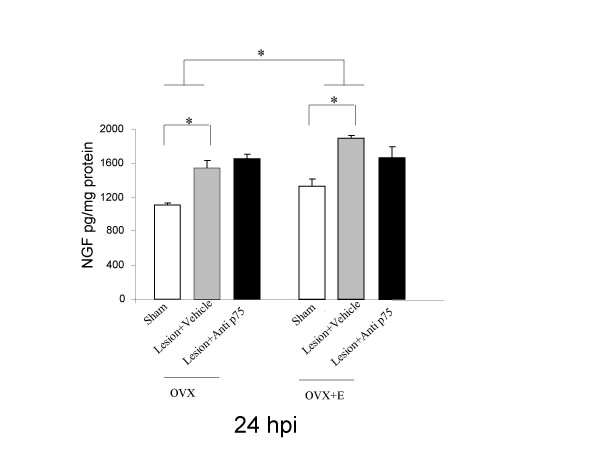
NGF regulation by lesion and estrogen treatment: Olfactory bulb lesions significantly increased NGF expression at 24 hours post injury (24 hpi). Estrogen treatment (OVX+E) increased basal and lesion-induced expression of NGF. However, anti-p75NTR had no effect on NGF expression in control pellet-replaced (OVX) or estrogen-replaced (OVX+E) animals. Bars represent means ± SEM, n = 6 per group. Sham: Sham lesion, Lesion+Vehicle: NMDA injection with pre immune serum, Lesion+Anti p75: NMDA injections with anti p75NTR antibodies. *:p < 0.05.

NGF expression in olfactory bulb lysates was measured by an ELISA assay and normalized to total protein. NGF is synthesized by a variety of cell types in the nervous and immune system and one of its principal actions in the brain is to promote growth and regeneration. As shown in Figure 8, olfactory bulb lesions increased NGF levels when measured at 24 h post lesion (F_2,30_: 24.154, p < 0.05) and estrogen treatment also increased constitutive and lesioned-induced expression of this growth factor (F_2,30_: 9.41, p < 0.05). However, anti-p75 antibodies did not attenuate the lesion-induced increase in NGF expression in either control or estrogen-treated animals, suggesting that the anti-p75NTR specifically targets the inflammatory cascade (IL-1β, PGE_2_, MMP-9) rather than repair pathways.

## Discussion

In previous studies, we have shown that the mechanical and excitotoxic damage caused by an NMDA injection to the olfactory bulb results in cell loss, astrocytosis and loss of cholinergic function [4], as well as microglial activation and a concomitant increase in the inflammatory cytokine IL-1β [5]. The present studies confirm our earlier observations that the inflammatory response in the forebrain of ovariectomized young adult rats is attenuated in animals that were replaced with estrogen as compared to animals that received a control (non-estrogen) pellet [5]. This report also shows that estrogen treatment further stimulates p75NTR expression, and that antibodies to p75NTR, administered concurrently with the lesion, completely abolish estrogen's anti-inflammatory effects. This study supports the hypothesis that estrogen's anti-inflammatory effects in the early portion of neural injury are mediated via the p75NTR. Since anti-p75NTR treatment had no effect on control replaced lesioned animals, these data further suggest that p75NTR expression in cells associated with the microvessel wall, which estrogen stimulates in lesioned animals only, may be a key cellular target of estrogen's anti-inflammatory effects.

The inflammatory cascade contains several protein and proteolipid mediators designed to contain the infectious agent and to phagocytize injured and dead cells. Mature IL-1β binds to its receptor and recruits the interleukin receptor associated kinase (IRAK), and this complex subsequently activates NFkB by association with TRAF-6, a member of the TNF receptor associated factor family [38,39]. Activation of signaling molecules, such as NFkB and JNK, result in the coordinate transcriptional expression of several downstream inflammatory mediators such as IL-6, IL-10, MMP-9 and cyclooxygenase-2 (COX-2), all of which contain motifs for NFkB or AP-1 in their promoter regions. The present study focused on a few key mediators, representing the early and later points of the inflammatory cascade. IL-1β, which is secreted by activated microglia/macrophages, is an early indicator of inflammation, and is known to activate JNK [40]. Both JNK and NFkB regulate MMP-9 [41] and COX-2 (the synthesizing enzyme for PGE_2_) [42]. In the present study, the lesion resulted in an early increase in IL-1β, JNK, and MMP-9 and a delayed increase in PGE_2_. All these indicators were attenuated in lesioned animals that received estrogen, and estrogen's actions were abrogated by anti-p75NTR antibodies. These data strongly support the hypothesis that p75NTR is an important mediator of estrogen's anti-inflammatory effects. While the mechanism of p75NTR anti-immune action is not yet known, one possibility is that p75NTR may interfere with IL-1β signaling pathways by competing for a limited pool of IRAK. NGF/p75NTR complexes are also capable of recruiting IRAK [32], and may therefore reduce the ability of interleukin receptor ligand complexes to activate NFkB or JNK, and consequently, hamper transcription of inflammation-related genes.

Identified as the first neurotrophin receptor [43,44], p75NTR has been implicated in both survival and apoptotic pathways [45]. While p75NTR interacts with the receptor tyrosine kinases, or trks, it has independent signaling activities mediated through the ceramide and JNK pathways as well [46]. P75NTR also has significant homology with a family of cell death molecules, such as TNFr and Fas (see [45,47] for reviews) and has been shown to promote apoptotic cell death in PC12 cells [48], retinal cells [49], oligodendrocytes [50], Schwann cells [51] and otic vesicles [52]. P75NTR expression also increases following injury, such as spinal cord injury [27,53]. More recently, studies have implicated p75NTR in the regulation of the inflammatory response, ranging from suppression of MHC molecules to preventing the transmigration of leukocytes. In hippocampal explant cultures, endogenous neurotrophins produced by neurons appear to control the antigen presenting ability of microglia, and neurotrophins, via p75NTR, suppress MHC II molecules [28]. P75NTR has also been localized to peripheral monocytes, and NGF, via p75NTR, prevents monocyte transmigration through the blood brain barrier [29]. In p75 knock-out animals, the severity of inflammation caused by a cranial nerve injury [54] or experimental allergic encephalomyelitis [24] is significantly greater as compared to wildtype controls. The p75NTR null animals had increased staining of activated microglia, and a massive recruitment of T-lymphocytes at the lesion site, indicative of increased permeability of the blood brain barrier [54]. The blood brain barrier serves to exclude peripheral cells, specific proteins and molecules from the brain, and p75NTR induction in endothelial cells following experimental allergic encephalomyelitis [24] suggests that this receptor may serve to maintain the integrity of the blood brain barrier. The present study indicates that estrogen exploits p75NTR-dependent anti-inflammatory mechanisms, and that this action may also occur at the blood brain barrier. In fact, estrogen itself is known to reduce transport across the blood brain barrier in injured [55,56] and non-injured animals [33] and reduces monocyte migration into the brain following ischemia [6].

Local levels of inflammatory cytokines such as IL-1β after traumatic neural injury are derived from two sources: initially from the activation of local microglia and eventually from the influx of circulating macrophages (and their products) that enter the brain due to progressive changes in the blood brain barrier. At 6 h after injury, estrogen treatment has a very modest effect on the IL-1β levels, and consequently, has little or no effect on JNK2 activation, which is activated by IL-1β/IL receptor complexes. At 24 h, however, estrogen markedly suppresses IL-1β levels and this is reflected in a pronounced decrease in JNK activation at this time point. This biphasic effect of estrogen on IL-1β suggests that estrogen may exert its effects not by acting on local microglia, but by reducing the pool of activated immune cells that are recruited to a central lesion site from circulatory sources, either by maintaining the integrity of the blood brain barrier, or by direct action on monocytes/macrophages. Our recent studies show that both possibilities are likely. For example, estrogen treatment suppresses cytokine production in circulating immune cells, but not microglia, when challenged *ex vivo *with LPS [57]. Furthermore, estrogen treatment also reduces permeability of the blood brain barrier as measured by extravasation of Evan's blue dye [33]. This latter hypothesis is also supported by the fact that estrogen fails to suppress IL-1β mRNA or the activity of caspase-1, which cleaves precursor IL-1β to its mature active form, at 24 hours post injury. This suggests that estrogen may not directly alter the availability of a local pool of IL-1β by canonical pathways but acts to gate the entry of cytokines from circulating immune cells or those produced by microvessel-associated cells.

Vascular permeability and leukocyte invasion can be increased by matrix remodelling resulting from the actions of the MMP family [58,59]. MMP-9 increases following experimentally induced stroke, and the availability of this proteinase directly contributes to the size of the infarct [60]. Several factors may control MMP-9 regulation and one report indicates that NGF associates with p75NTR to downregulate MMP-9 activity [61]. Estrogen also has been shown to reduce MMP-9 mRNA [6,62] which likely contributes to its neuroprotective actions. In the present study, estrogen-induced decreases in MMP-9 were reversed by anti-p75NTR. Although this study does not address the source of MMP-9 or its substrate, it may be the case that estrogen-induced reductions in MMP-9 provide the means for maintaining the integrity of the blood brain barrier in these animals.

The observation that estrogen treatment exacerbates p75NTR expression in lesioned animals is surprising in view of the many studies where estrogen has been shown to decrease p75NTR mRNA [63-65] and protein [66,67] expression. However, these studies were performed in unlesioned animals (without neural trauma), and may therefore be representative of neuronal p75NTR, as has been shown in basal forebrain neurons [65]. This was also the case in a more recent study where estrogen suppressed p75NTR expression in hippocampal neurons following ischemic injury in male gerbils [68]. Moreover, the same study also reported that p75 expression in the hippocampus was first seen 48 h after injury. The present study, on the other hand, describes a very early event in the injury process. Furthermore, the only region where p75NTR expression was regulated differentially was in the microvasculature of estrogen-treated, lesioned animals. This specificity may explain why anti-p75NTR treatment abolished estrogen's effects on the inflammatory cascade but had no discernable effects on the placebo-replaced animals. We hypothesize that the early increase in microvascular p75NTR may play a significantly different role in inflammation as compared to the later expression of neuronal p75NTR.

The present study shows that estrogen replacement to ovariectomized young females suppresses the inflammatory response following neural injury; however, hormonal regulation of the inflammatory response is not always benign. While estrogen reduces experimentally induced inflammation in the anterior chamber of the eye [69], lungs [70] and the tibiotarsal joint in adjuvant-induced arthritis [71,72], this hormone promotes inflammation in the prostate [73,74], and stimulates edema [75], vascular permeability and influx of macrophages in the uterus [76-79]. Even in models of neurogenic inflammation, such as the present study, estrogen treatment is not uniformly neuroprotective. For example, a similar 3 wk regimen of estrogen to reproductive senescent females does not attenuate the production of IL-1β, and actually exacerbates it [5]. Furthermore, in these senescent animals, estrogen fails to enhance p75NTR expression following a lesion (data not shown). While the reasons for estrogen's disparate actions on different tissues is not clear, the present study suggests that p75NTR may be one of the switches that predict whether estrogen will exert neuroprotective or neurotoxic effects. An important direction for this work would be to test this hypothesis in the p75 knock-out model or, preferably, a conditional p75NTR knock-out animal, which eliminates the compensatory changes that might occur as a result of developmental loss of p75.

## Methods

### Animals

Sprague Dawley females (~250 g, 4 months) were purchased from Harlan Laboratories (IN) and maintained in an AALAC-approved facility on a 12-h light: 12-h dark cycle with lights on at 06:00 h, with food and water available *ad libitum*. All procedures were in accordance with NIH and institutional guidelines governing animal welfare. Several sets of animals were prepared, each with internal controls. For convenience a chart is provided below. For the p75 expression studies (Westerns blot analysis and immunohistochemistry), we used tissue from four sets of animals that had been prepared for a previous study, and related data from these animals is published in [5]. Briefly, young adult animals were ovariectomized and replaced with either control or estrogen pellets and 3 weeks later were assigned to either sham lesion or NMDA lesion groups. Twenty four hours later, animals were sacrificed and the olfactory bulbs were dissected and later harvested for proteins. A second set was prepared as above for histological analysis, where animals were perfused following anesthetic overdose and the brains recovered for histological processing (described later).

For p75NTR antibody studies, two sets of animals were prepared: placebo or estrogen-replaced animals were assigned to one of three groups: sham lesion, NMDA+vehicle control (preimmune rabbit serum) or NMDA+anti-p75NTR antibodies (made in rabbit). One set of animals was sacrificed at 6 h after the lesion, and the second set at 24 h after the lesion. In all sets, each treatment group consisted of 5-6 animals per group (See Table 1). Specific surgical procedures are detailed below.

Animals were also used for pilot studies to (a) determine the most effective concentration of anti-p75NTR antibodies (n = 12) and (b) to ensure that there were no differences between animals injected with NMDA and NMDA+preimmune serum (the vehicle for the anti-p75NTR antibody) (n = 6).

### Surgical techniques

Ovariectomies: As previously described [4,66,80,81], animals were anesthetized with ketamine (87 mg/kg)/xylazine (13 mg/kg) and bilateral ovariectomies were performed using a dorsal midline incision inferior to the palpated rib cage and kidneys. Ovaries and surrounding tissue were removed and 60-day time-release 17β estradiol pellets (1.0 mg) or control pellets (Innovative Research, FL) were inserted subcutaneously (s.c.) prior to closing the incision. These pellets have been used extensively in our work and have resulted in physiological levels of plasma estradiol for periods of 3 to 6 weeks [4,5,66].

### Stereotaxic surgeries

After 3 weeks of estrogen or control treatment, all animals were anesthetized with ketamine (87 mg/kg)/xylazine (13 mg/kg) and placed in a rodent stereotaxic apparatus. Skin and cranial fascia were resected and the skull exposed. Two small craniotomies were made in all rats assigned to lesion groups, to expose the olfactory bulbs at the following coordinates: 7.6 mm anterior to bregma and 1.0 mm lateral to the sagittal suture. The tip of a Hamilton syringe needle was briefly lowered to a depth of 3.4 mm, and immediately raised by 0.2 mm to create a trough. A total of 2 μl (1 μl per side) of 50 nM NMDA was injected at a rate of 0.2 μl/30 s. For anti-p75NTR experiments, 1 μL of 50 nM NMDA + 2 μL preimmune rabbit serum (L groups) or 1 μL 50 nM NMDA + 1.5 μL preimmune rabbit serum + 0.5 μL ap75NTR (P groups) was injected in each bulb at a rate of 0.5 μL/30 s. After injections, the needle was raised slowly, craniotomies filled with gel-foam, and scalp sutured with wound clips. Surgical controls were anesthetized, restrained in the stereotaxic apparatus and their scalp and cranial fascia resected and reclipped. Other reports [82] and our previous studies [5] indicate that at 24 h post injury a saline injection is not an appropriate control since the injection itself results in injury.

The anti-p75NTR antibody used here (Chemicon, CA, AB1554) recognizes the extracellular domain of the p75NTR and has been shown to block NGF/p75NTR interactions [83-85]. This antibody is not recommended for Western blots (hence a different antibody was used for the Western blot assays), but can be used for immunohistochemistry. Both the neutralizing antibody used here and the antibody used for Westerns revealed similar staining patterns in the olfactory bulb when used for immunohistochemistry.

Animals were sacrificed at 6 h and 24 h after stereotaxic surgery by rapid decapitation and trunk blood was collected for estimation of estradiol content by radioimmunoassay (Diagnostic Systems Laboratories, TX). Olfactory bulbs were rapidly removed and stored at -80°C. Proteins were isolated from the entire olfactory bulb using previously established procedures [4,80,81] and total protein concentrations were determined using the BCA protein assay kit (Pierce, IL). In some cases, bulbs were processed for RNA extraction described below.

### Western blot analysis

Equal amounts of total protein from tissue lysates were size-fractionated on a polyacrylamide gel, transferred to a nylon membrane (Hybond C-Super, Amersham, NJ) and analyzed for p75NTR and pJNK. Blots were also probed for total JNK expression as a loading control, since this protein did not vary with lesion or estrogen treatment [81]. Membranes were blocked with 5% milk in 1 × Tris Buffered Saline + 0.05% Tween (TTBS) solution for 1 h, followed by incubation with the primary antibody in milk-TTBS (1/ 1500 Ms × NGF Receptor (p75NTR; MAB365), Chemicon, CA; 1/2500 anti-active JNK, Promega, WI; 1/2000 JNK, Santa Cruz, CA) for 1 h at room temperature (p75NTR) or overnight at 4°C (pJNK, JNK). Blots were washed with TTBS (3 × 10 min) and primary antibody was detected using an HRP-conjugated secondary antibody in milk-TTBS (anti-mouse IgG 1/3000 for p75NTR, Transduction Laboratories, KY; 1/6000 anti-rabbit IgG for pJNK, Promega, WI; 1/2000 anti-rabbit IgG for JNK, Santa Cruz, CA). All incubations were performed at room temperature with gentle shaking. After the last wash step, an enzyme-catalyzed chemiluminescent reagent (Renaissance NEN, MA) was used for immunodetection. Signals were detected using X-ray film and bands quantified by densitometric analysis (Molecular Analyst, Bio-Rad, CA) and normalized to JNK expression.

### Immunohistochemistry for p75NTR

Immunohistochemistry was performed on olfactory bulb sections of placebo- or estrogenreplaced ovariectomized young-adult animals that were subject to olfactory bulb lesions as outlined above and sacrificed 24 h later. Sections were prepared using the NeuroScience Associates Multibrain technology and some sections from this set were used in previous assays to detect activated microglia [5]. Briefly, animals were anesthetized with 0.6 mL pentobarbital solution (50 μg/mL pentobarbital, 10% ethanol, 40% propylene glycol) and perfused transcardially with PBS followed by 4% paraformaldehyde. Brains were removed from the skull and post-fixed in 4% paraformaldehyde (2 h). Brains were then shipped to NeuroScience Associates (Knoxville, TN), where they were treated with 20% glycerol and 2% dimethylsulfoxide to prevent freeze artifacts, and then embedded coronally in groups of 14-16 per block in a gelatin matrix. 40 μm freeze-cut sections through the entire olfactory bulbs were obtained with an AO 860 sliding microtome and collected in 4 × 6 array of containers filled with 10% phosphate-buffered formaldehyde. After 24 h, sections were rinsed and transferred into Antigen Preserve solution (50% PBS pH 7.0, 50% Ethylene glycol, 1% PVP) for storage and shipping. Prior to use in immunohistochemistry, sections were rinsed in PBS and mounted on gelatin coated glass slides. P75NTR was detected by fluorescence-labeled secondary. Sections were incubated with block solution and followed by overnight incubation with the primary antibody (1/200; mouse anti-NGFR; Chemicon, CA) diluted in PBS with 3% goat serum and 0.4% Triton. Controls were incubated with diluent only. Following washes, sections were incubated with a rat-adsorbed FITC-conjugated Fab specific secondary antibody (1/160 goat anti-mouse, Vector Labs, CA)or AlexaFluor 488 (1:2000 for goat anti-mouse, Invitrogen, CA) for 45 min at room temperature. Sections were then washed and coverslipped using a fluorescence-compatible mounting media which contained DAPI, a nuclear dye. The presence of glomerular staining in the bulb was also used as a positive control. Sections from the midpoint of the bulb (around the injection site) were examined under fluorescence illumination for FITC for the presence of p75NTR immunostaining. Five sections were examined from each animal and 5-6 animals were examined per treatment group.

### Caspase-1 and Caspase-3 assays

A commercial kit was used to determine Caspase-1 (interleukin-1 converting enzyme) and Caspase-3 activity (Promega, WI) as before [81], using manufacturer's recommended procedures. Briefly, blanks, samples and negative controls were pipetted into a flat-bottom, black 96-well plate and incubated at 30°C for 30 minutes. Plates were then incubated with Caspase-1 (ICE) or CCP32 substrate (Ac-YVAD-AMC and Ac-DEVD-AMC, respectively) at 30°C for 60 minutes. A standard curve was prepared just prior to reading at 360 nm (excitation) and 460 nm (emission) in a fluorescence microplate reader (BioTek, VT). Measurement of liberated AMC, interpolated from standard curves, was normalized to protein per unit time (min).

### Enzyme-linked immunosorbent assay (ELISA)

Commercial kits for IL-1β (R&D Systems, MN) and PGE_2 _(Cayman Chemical, MI) were used to determine cytokine and prostaglandin levels in tissue lysates, using procedures recommended by the manufacturers [5]. Briefly, standards, controls, samples and a biotinylated (IL-1β) or acetylcholinesterase-linked (PGE_2_) secondary antibody were pipetted into 96-well plates pre-coated with antibodies specific for rat IL-1β and PGE_2_, and incubated at RT for 2 h (IL-1β) or 4°C overnight (PGE_2_). Following washes, plates were sequentially incubated with streptavidin peroxidase (IL-1β: 2 h) or Ellman's reagent (PGE_2_: 60-90 min) and substrate solution for 30 min (IL-1β). Plates were read at 450 nm (IL-1β) or 405 nm (PGE_2_) in a microplate reader (Bio-Tek, VT). Standard curves were established from optical densities of wells containing known dilutions of standard, using KC3 software (Bio-Tek, VT) and sample measurements were interpolated from standard curves.

### Reverse transcription-polymerase chain reaction

IL-1β and MMP-9 gene expression was determined by semi-quantitative reverse transcription-polymerase chain reaction (RT-PCR). RNA was extracted using the TRIZOL^® ^Reagent RNA extraction method [86], followed by further purification with the Qiagen RNeasy Kit (Qiagen, CA). The RNA concentration was determined using the RiboGreen^® ^RNA Quantification Kit (Molecular Probes, OR). Reverse transcription of total RNA (2 μg) was performed with the Gibco Superscript™ First Strand Synthesis System. PCR of the newly synthesized cDNA (8 ng) was performed using PCR Supermix (Invitrogen, CA; primer conc. 0.25 μM; 200 μM dNTP; 1× PCR Buffer, 1.5 mM MgC1_2_, 20 U recombinant Taq DNA polymerase). IL-1β (5'-3': IL-1β-For TTG AAT CTA TAC CTG TCC TGT GTG; IL-1β Rev TGA CTT GGC AGA GGA CAA AGG) PCR cycles were as follows: 95°C 2 min; 30 cycles of 95°C 30 sec, 60°C 1 min, 72°C 2 min. PCR reactions for MMP-9 were performed using previously published primers [11]. PCR conditions were as follows: 95°C for 5 min followed by 40 cycles at 92°C for 1 min, 56°C for MMP-9 for 1 min, and 72°C for 1 min. Cyclophilin (5'-3': CPI-1, TGG TCA ACC CCA CCG TGT TCT TCG; CP1-2 TGC CAT CCA GCC ACT CAG TCT TGG) was used to normalize IL-1β and MMP-9 expression. The PCR cycles for cyclophilin are as follows: 95°C 2 min, 20 cycles of 95°C 30 sec, 62°C 1 min, 72°C 2 min. PCR reactions were performed on a Perkin-Elmer Thermal Cycler 480. Initial analyses were performed to ensure that IL-1β and cyclophilin were amplifying in the linear range. The PCR reaction was separated on a 1.5% agarose gel and quantified using Molecular Analyst™ (BioRad, CA). To avoid quantitation artifacts, only samples loaded on the same gel were analyzed. Hence, for each treatment group, 4 independent samples were analyzed. In every case, only a single band of the expected size was detected and bands amplified by each primer set was sequenced (Gene Technologies Laboratory, TAMU) and determined to be homologous to rat IL-1β and MMP-9 gene sequences.

### Gelatin zymography

Procedures used here are a modification of our previous protocol using culture media [57]. Equal amounts of total protein were size fractionated on a 10% polyacrylamide gel containing 0.01% gelatin, along with prestained protein size markers. On some gels, a positive control was included (conditioned media from human umbilical vein endothelial cells; kind gift of G.E. Davis, TAMUS HSC). After electrophoresis, the gels were rinsed with MQ water and then incubated with renaturing buffer (2% Triton X-100 in MQ) at room temperature for 1 h with 3 buffer changes. Gels were then rinsed 3× with MQ water and incubated with developing buffer (50 mM Tris, 0.2 M NaCl, 5 mM CaCl_2 _0.02% Brij 35) overnight at 25°C with gentle shaking. After a brief rinse with MQ water, gels were stained with a 0.25% coomassie blue solution (50% methanol, 20% acetic acid) for 1 h and destained in 20% methanol: 10% acetic acid. Gels were dried at 50°C for 1 h, and later digitized. A standard densitometric program (Quantity One, BioRad, CA) was used to calculate the intensity of the lytic area.

### Statistical analysis

Statistical analysis was performed using a statistical software package (SPSS Inc, IL), and group differences were considered significantly different at p < 0.05. Data was subject to a two-way analysis of variance (ANOVA) with hormone and lesion as independent variables. Planned post-hoc comparisons were performed for the lesion variable in those studies where there were three groups (sham, lesion+vehicle (preimmune serum) and lesion+anti-p75NTR).

## Abbreviations

ERK: Extracellular-signal regulated kinase; ICE: Interleukin-1 converting enzyme; IL-1β: Interleukin-1beta; IRAK: Interleukin receptor-associated kinase; JNK: C-jun terminal kinase; LPS: Lipopolysaccharides; MMP-9: Matrix metalloproteinase-9; NFkB: Nuclear factor-kappaB; NGF: Nerve Growth factor; NMDA: N-methyl-d-aspartate; P75NTR: P75 neurotrophin receptor; PGE_2_: Prostaglandin E_2_, PI-3K: Phosphatidylinositol-3 kinase; TNFR: Tumor necrosis factor receptor; TRAF: TNFR associated factor

## Authors' contributions

VLN performed almost all experiments and prepared most figures, DKL performed RT-PCR analyses and prepared the associated figures, SB participated in data analysis and interpretation, FS conceived the study, performed statistical analysis and photomicroscopy. All authors read and approved the final manuscript.

## Figures and Tables

**Table 1:** 

**Summary**	**P75NTR Western**		**P75NTR Immuno**		**RNA analysis**		**Antip75 Expt 6 h**			**Antip75 Expt 24 h**		
	
	**Sham Lesion**	**NMDA**	**Sham Lesion**	**NMDA**	**Sham Lesion**	**NMDA**	**Sham Lesion**	NMDA+ Veh*	**NMDA+Anti p75NTR**	**Sham Lesion**	NMDA+ Veh*	**NMDA+Anti p75NTR**
**Control Pellet**	6	6	5	6	6	6	6	6	6	6	6	6
**Estrogen-Pellet**	6	6	6	6	6	6	6	6	6	6	6	6

**Analysis**	**Western blots**		**Immuno histochemistry**		**RT-PCR analysis of IL-1β, MMP-9**		**IL-1β, pJNK, PGE2, MMP-9,**			**IL-1β, pJNK, PGE2, MMP-9 caspase-3, caspase-1**		

* = Pre immune serum
